# Why we need to pursue both universal and targeted prevention to reduce the incidence of affective and psychotic disorders: Systematic review and meta-analysis

**DOI:** 10.1016/j.neubiorev.2024.105669

**Published:** 2024-06

**Authors:** Sebastien Brodeur, Dominic Oliver, Muhammad S. Ahmed, Joaquim Radua, Jemma Venables, Yueming Gao, Vincenzo Gigante, Giulia Veneziano, Giulia Vinci, Edward Chesney, Sunil Nandha, Andrea De Micheli, Ilaria Basadonne, Valentina Floris, Gonzalo Salazar de Pablo, Paolo Fusar-Poli

**Affiliations:** a Early Psychosis: Interventions and Clinical-Detection (EPIC) Lab, Department of Psychosis Studies, Institute of Psychiatry, Psychology & Neuroscience, King's College London, London SE5 8AF, UK; b Département de Psychiatrie et Neurosciences, Université Laval, Canada; c Department of Psychiatry, University of Oxford, Oxford OX3 7JX, UK; d NIHR Oxford Health Biomedical Research Centre, Oxford OX3 7JX, UK; e OPEN early detection service, Oxford Health NHS Foundation Trust, Oxford OX3 7JX, UK; f South London and Maudsley NHS Foundation Trust, London, UK; g Institut d’Investigacions Biomèdiques August Pi i Sunyer, University of Barcelona, Barcelona, Spain; h CIBERSAM, Instituto de Salud Carlos III, Madrid, Spain; i Department of Psychiatry, University of Cambridge, Cambridge, UK; j Department of Brain and Behavioral Sciences, University of Pavia, Pavia, Italy; k Department of Psychosis Studies, Institute of Psychiatry, Psychology & Neuroscience, King's College London, London SE5 8AF, UK; l OASIS Service, South London and Maudsley NHS Foundation Trust, London SE11 5DL, UK; m Department of Child and Adolescent Psychiatry, Institute of Psychiatry, Psychology & Neuroscience, King's College London, London, UK; n Child and Adolescent Mental Health Services, South London and Maudsley NHS Foundation Trust, London, UK; o Department of Psychiatry and Psychotherapy, Ludwig-Maximilian-University Munich, Germany

**Keywords:** Psychosis, Bipolar, Depression, Prevention, Universal, Intervention, Meta-analysis

## Abstract

The effectiveness of universal preventive approaches in reducing the incidence of affective/psychotic disorders is unclear. We therefore aimed to synthesise the available evidence from randomised controlled trials. For studies reporting change in prevalence, we simulated all possible scenarios for the proportion of individuals with the disorder at baseline and at follow-up to exclude them. We then combined these data with studies directly measuring incidence and conducted random effects meta-analysis with relative risk (RR) to estimate the incidence in the intervention group compared to the control group. Eighteen studies (k=21 samples) were included investigating the universal prevention of depression in 66,625 individuals. No studies were available investigating universal prevention on the incidence of bipolar/psychotic disorders. 63 % of simulated scenarios showed a significant preventive effect on reducing the incidence of depression (k=9 – 19, RR=0.75–0.94, 95 %CIs=0.55–0.87,0.93–1.15, p=0.007–0.246) but did not survive sensitivity analyses. There is some limited evidence for the effectiveness of universal interventions for reducing the incidence of depression but not for bipolar/psychotic disorders.

## Highlights

• No evidence for universal prevention for bipolar/psychotic disorders.
• Limited evidence for universal prevention for depression.
• Universal prevention can complement selective and indicated approaches.
• Small effect sizes are expected so high quality, well-powered studies are needed.

## 1 Introduction

Affective and psychotic and disorders have a significant impact and on individuals, their families and society (Estradé et al., 2023, Fusar-Poli et al., 2023, Fusar-Poli et al., 2022), leading to various preventive approaches being developed (Fusar-Poli et al., 2021). The World Health Organisation (WHO) has defined three types of preventive intervention: primary (aiming to prevent the onset of an illness/disorder, i.e. incidence), secondary (to reduce the number of cases of an illness/disorder, i.e. prevalence, through treatment at the first episode stage) and tertiary prevention (rehabilitation, relapse prevention, etc.) (Fusar-Poli et al., 2021, WHO, 2004). Despite significant progress in improving patients’ symptoms with secondary and tertiary interventions, reducing the incidence of affective and psychotic disorders through primary prevention remains a clear goal (Fusar-Poli et al., 2021). Primary prevention can be further divided into three types of intervention: universal, selective and indicated, defined by the Gordon Classification (Institute of Medicine Committee on Prevention of Mental, 1994). Universal prevention is a population-level approach, aiming to reduce exposure to risk factors or increase its exposure to protective factors (Fusar-Poli et al., 2021). Targeted approaches include selective prevention in subgroups with specific risk (for example, children of parents with bipolar disorder or schizophrenia) and indicated approaches in people who experience attenuated symptoms (Arango et al., 2021, Fusar-Poli et al., 2021, Uher et al., 2023).

There is high potential for universal preventive interventions in mental health, due to the large scale and relatively high acceptability of low intensity interventions (Pedrini et al., 2022). This potential has led some researchers to argue that universal interventions may be more effective than targeted (selective or indicated) interventions.

However, the effectiveness of available universal interventions for reducing the incidence of affective and psychotic disorders is unknown (Fusar-Poli et al., 2017). Previous research in this area has largely focused on depression (Cuijpers et al., 2021, Hoare et al., 2021, Rigabert et al., 2020). However, it has largely focused on severity of depressive symptoms (Hoare et al., 2021), which does not automatically translate into reduced incidence. An umbrella review showed that school-based and e-health interventions may provide some evidence for the prevention of depression (Hoare et al., 2021). However, this study assessed the effect of these interventions on reducing the severity of depressive symptoms, rather than the incidence of depression. It therefore is difficult to conclude whether these universal preventive interventions are effective at reducing the incidence of depression, which is truly reflective of preventing depression. Indeed, numerous previous studies have used scales measuring severity of psychiatric symptoms in large samples, but ultimately the goal of preventive psychiatry in this context is not to reduce the average severity of subthreshold psychiatric symptoms, it is to prevent the onset of a full threshold diagnosis of an affective or psychotic disorder. Therefore, our study will be restricted to assessing the effect of interventions on reducing the incidence of affective and psychotic disorders. Because the effect sizes are expected to be small due to the relatively high numbers needed to treat to prevent a case (Salazar de Pablo et al., 2021), it is essential to focus on the highest quality evidence from randomised controlled trials.

The aim of the present study is therefore to systematically examine randomised controlled trials of universal preventive interventions for affective and psychotic disorders.

## 2 Methods

This systematic review, compliant with the Preferred Reporting Items for Systematic Reviews and Meta-analyses 2020 (PRISMA 2020; Table 1) (Page et al., 2021) and Meta-analyses of Observational Studies in Epidemiology (MOOSE; eTable 2) (Stroup et al., 2000) checklists, was registered on PROSPERO (CRD42023428746). We followed EQUATOR Reporting Guidelines (Altman et al., 2008).

**Table 1:** Characteristics of the included studies.

**Study**	**Intervention Type**	**Trial Type**	**Sample Size**	**Setting**	**Target**	**Age (mean)**	**Gender** **(% Male)**	**Instrument**	**Intervention Duration**	**Follow-up Time**
**Depression (Incidence)**										
(Berk et al., 2020)	Pharmacological	Parallel	18,702	Primary care	Older adults	75.2	43.6	Self-report	6 years	6 years
(Calear et al., 2009)	Psychological – individual, virtual	Cluster	1,477	School	Adolescents	14.3	44.0	Self-report	5 weeks	6 months
(Imamura et al., 2015)	Psychological – individual, virtual	Parallel	762	Workplace	Adults	37.6	83.9	Diagnostic interview	6 weeks	1 year
(Muñoz et al., 1995)	Psychological – individual, in person	Parallel	150	Primary care	Adults	52.5	38.0	Diagnostic interview	8 weeks	1 year
(Rooney et al., 2013)	Psychological – group, in person	Cluster	910	School	Children	8.8	51.4	Diagnostic interview	10 weeks	18 months
(Rose et al., 2014)	Psychological – group, in person	Cluster	210	School	Adolescents	12.2	56.0	Self-report	9 weeks	1 years
(Whittaker et al., 2017)	Psychological – individual, virtual	Parallel	855	School	Adolescents	14.3	31.7	Diagnostic interview	9 weeks	1 year
**Depression (Prevalence)**										
(Almeida et al., 2012)	Psychoeducation	Cluster	21,762	Primary care	Older adults	71.8	41.2	Self-report	2 years	2 years
(Bond et al., 2004)	Psychological – group, in person	Cluster	2,678	School	Adolescents	NA	47.0	Diagnostic interview	10 weeks	4 years
(Carta et al., 2022)	Exercise	Parallel	120	Community	Older adults	72.4	54.9	Self-report	12 weeks	4 years
(Chen et al., 2022)	Psychological – group, in person	Cluster	316	School	Children	10.1	47.8	Self-report	8 weeks	1 year
(Eyigor et al., 2009)	Exercise	Parallel	37	Community	Older adults	72.4	0	Self-report	8 weeks	8 weeks
(Heckendorf et al., 2022)	Psychological – individual, virtual	Parallel	351	Internet	Adults	42.6	17.1	Self-report	6 months	2 weeks
(Komariah et al., 2022)	Psychological – group, virtual	Parallel	122	University	Adults	20.3	23.8	Self-report	4 weeks	4 weeks
(Montero-Marin et al., 2022)	Psychological – group, in person	Cluster	8,376	School	Adolescents	12.2	43.2	Self-report	1 year	1 year
(Perry et al., 2017)	Psychological – individual, virtual	Cluster	540	School	Adolescents	16.7	36.8	Self-report	1 week	18 months
(Prencipe et al., 2022)	Economic	Cluster	2,458	Community	Adolescents	16.0	55.0	Self-report	1 year	1 year
(Teesson et al., 2020)	Psychological – group, virtual	Cluster	6,386	School	Adolescents	13.5	45.2	Self-report	12 weeks	30 months

### 2.1 Search strategy

Ovid (MEDLINE, EMBASE and PsycINFO) database was searched from inception to 23rd May 2023.

The search strategy was: (prevent* OR population-level OR universal OR public health) AND (psychosis OR psychot* OR psychological OR depress* OR bipolar OR schizo* OR affective OR mood OR mental) AND (randomised controlled trial OR randomized controlled trial OR randomi?ed* OR RCT OR trial)

In addition to searching databases, the authors examined the reference lists of included articles and relevant identified reviews. Duplicate articles were first identified automatically, then manually.

An independent, two-step literature search was conducted. Two investigators (from SB, DO, MSA, JV, YG, VG, GV and GV) assessed article inclusion by examining titles and abstracts sequentially in parallel, blinded to each other’s ratings. Where there was disagreement between reviewers, the study in question was included for the next round of screening. Articles identified for full-text screening were retrieved by searching online catalogues and university libraries. Where articles could not be retrieved, the authors were contacted with a request to provide the text. Two of the authors (SB, DO, MSA, JV, YG, VG, GV and GV) assessed article inclusion by examining the full texts of the identified articles in parallel, blinded to each other’s ratings. Any discrepancies were resolved in consensus meetings with two of the authors (DO, PFP).

### 2.2 Selection criteria

Inclusion criteria were: (i) original parallel-group or cluster randomised controlled trial of a universal preventive intervention of depression, bipolar disorder or psychosis with an intervention and control group (randomised and employing either a double-blind, single-blind; open-label; parallel group or cross-over design); (ii) published in a peer-reviewed journal in English or Italian; (iii) investigating the follow-up incidence of affective and psychotic disorders in samples without these disorders at baseline, or the prevalence of affective and psychotic disorders at follow-up compared again to baseline; (iv) reporting data that could be meta-analysed (number of events, odds ratios, or risk ratios).

Exclusion criteria were: (i) reviews, conference proceedings, or pilot data; (ii) target population selected on the basis of increased risk of developing depression, bipolar disorder or psychosis (i.e. selective intervention) or presenting with subthreshold conditions, clinical or subclinical (i.e. indicated intervention); (iii) written in languages other than English and Italian; (iv) overlapping samples; (v) lack of control group; (vi) not assessing an affective or psychotic disorder; (vii) not reporting required outcome data for meta-analysis. We searched for overlap by looking at the name of the preventive program or the type of the intervention as well as the population and location in which the study was carried out. In case of overlap, we solely included the largest and most representative study and excluded the others.

### 2.3 Data extraction

The researchers independently extracted data from all included studies. Both of the resulting databases were then crosschecked by experienced researchers (SB, DO), and discrepancies were resolved through consensus. Variables included in the study were the following: authors and years of the study; country; intervention type (economic, exercise, pharmacological, psychoeducation, psychological group in person, psychological group virtual, psychological individual in person, psychological individual virtual); sample size; gender (% male); age (mean age; SD or range if mean was not available); intervention duration; follow-up time; assessment instrument; target population and target disorder.

### 2.4 Risk of bias

The Cochrane Risk of Bias 2 tool (Sterne et al., 2019) for parallel-group and cluster-randomised trials were used to assess and classify the risk of bias in each of the included studies, as per criteria defined *a priori*. These criteria were clustered into the following domains: randomisation, deviations from the intended intervention, missing outcome data, outcome measurement and selection of reported result.

### 2.5 Statistical analysis

For studies reporting prevalence at baseline and follow-up (i.e., before and after the intervention in both the intervention and control group), we could not use conventional meta-analysis to estimate between-group differences in incidence. This was due to an unknown combination of individuals with the outcome of interest at baseline who either dropped out or remitted over the course of follow-up leading to negative within-group changes in prevalence in some cases.

**Fig. 1:**
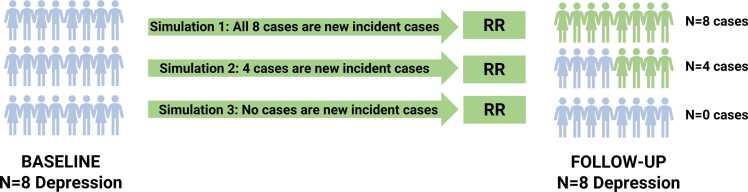
Overview of simulation approach for meta-analysis for the primary outcome. We know the number of individuals with the disorder at baseline, who should be excluded, and while we know the number of individuals with the disorder at follow-up, we do not know if any of these are the same individuals with the disorder at baseline. Therefore, we tested all combinations of the percentage of individuals with a disorder at baseline who have not dropped out of the study and still meet criteria at follow-up. This allowed us to conduct a meta-analysis of incident cases, combined with studies that directly measured incidence, and a relative risk was calculated.

To model incident diagnoses in these studies, we had to make assumptions regarding how many cases at follow-up were also recorded as cases at baseline to exclude them from the analysis. Therefore, we used a simulation approach (Fig. 1), simulating the proportion of cases at baseline who were also recorded as cases at follow-up (i.e. those who did not drop out or remit over the course of follow-up from 0 % to 100 %) and should therefore be excluded from the analysis. Following these steps, the resulting number of individuals were subtracted from the follow-up cases to be considered as the number of incident cases. For each simulation, we excluded studies with impossible scenarios (e.g., if there was a study with five individuals with the disorder at baseline and ten individuals with the disorder at follow-up, a minimum of five incident cases and a maximum of ten incident cases over the study period would be considered to be feasible). We then combined these data with data from studies comparing incidence risk. As these studies are directly measuring the outcome of interest and are more reliable, we re-scaled our data from studies reporting prevalence to be equivalent to the sample size of studies comparing incidence risk. With this combined dataset, we conducted a random-effects meta-analysis. The effect size measures were relative risk (RR) with 95 %CIs. Values less than 1 indicated a greater preventive effect in the intervention group versus the control group. We present the range of meta-analytic estimates across all possible scenarios.

Our primary sensitivity analysis restricted our meta-analysis to studies comparing incidence risk. We then stratified for the type of intervention provided, intervention target size (individual or group) and intervention delivery format (in person or virtual) across all studies. Analyses restricted to studies employing clinical interviews to measure the primary outcome (affective or psychotic disorder onset) were also performed. Heterogeneity among study point estimates was assessed using the Q statistic. The proportion of the variability in effect size estimates due to between-study heterogeneity was evaluated using the I^2^ index (I^2^>50 % represents substantial heterogeneity) (Lipsey and Wilson, 2001). When more than nine studies were included in an analysis, we assessed publication bias using Egger’s test (Egger et al., 1997) and the ‘trim and fill’ method (Duval and Tweedie, 2000) to correct for the presence of missing studies when a risk of publication bias was detected.

Data were analysed using R (version 4.2.2) and the meta package (version 6.1–0). The threshold for significance was set to p < 0.05.

## 3 Results

**Fig. 2:**
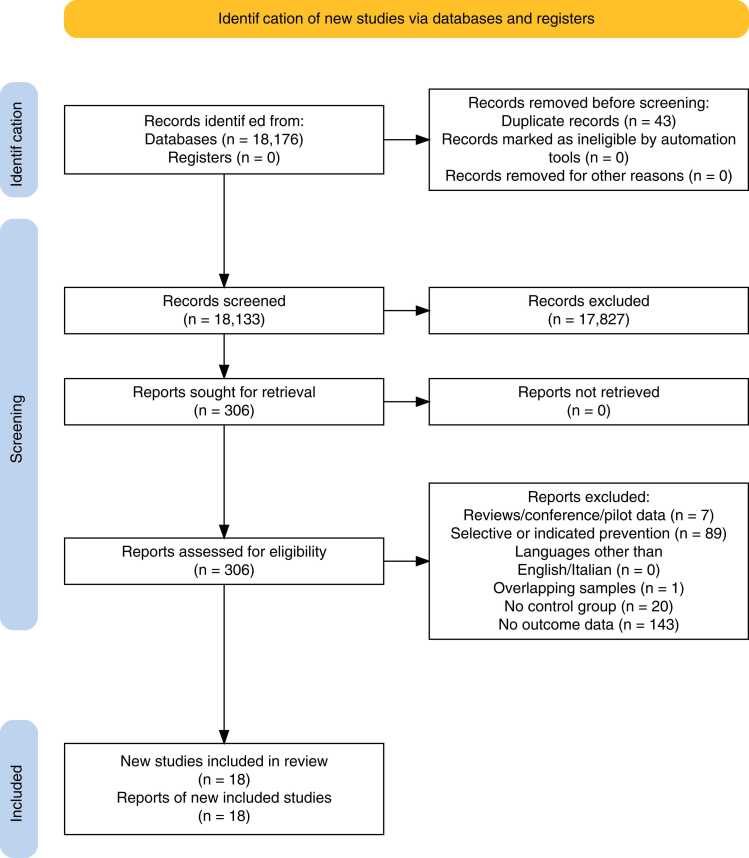
Study selection.

Overall, 18,176 records were searched, 43 duplicates were removed, 1282 abstracts were screened, 306 full texts were screened and 18 were eligible (Fig. 2, Table 1). Overall, the 18 eligible studies, comprising k=21 samples and 66,625 patients, reported on randomised controlled trials of universal interventions to prevent affective/psychotic disorder onset. All 18 studies reported data on universal preventive interventions for depression, with no studies reporting data on universal preventive interventions for bipolar disorder or psychotic disorders. Universal preventive interventions were stratified for descriptive purposes into eight categories: psychological - group, in person (5 studies), psychological - individual, virtual (5 studies), exercise (2 studies), psychological - group, virtual (2 studies), economic (1 studies), pharmacological (1 studies), psychoeducation (1 studies) and psychological - individual, in person (1 studies) for an overall average 2.93 years follow-up (sample size-weighted mean).

### 3.1 Primary meta-analysis

Eleven studies (k=14 samples) reported data on the pre/post prevalence of depression (k=5 psychological - group, in person; k=4 psychological - group, virtual; k=2 exercise; k=1 economic; k=1 psychoeducation; k=1 psychological - individual, virtual). Seven studies (k=7 samples) reported data on the incidence of depression (k=4 psychological - individual, virtual; k=1 pharmacological; k=1 psychological - individual, in person; k=1 psychological - group, in person).

92 (92 %) of the simulated scenarios resulted in sufficient studies with non-negative numbers of cases and non-cases, which enabled meta-analysis.

**Fig. 3:**
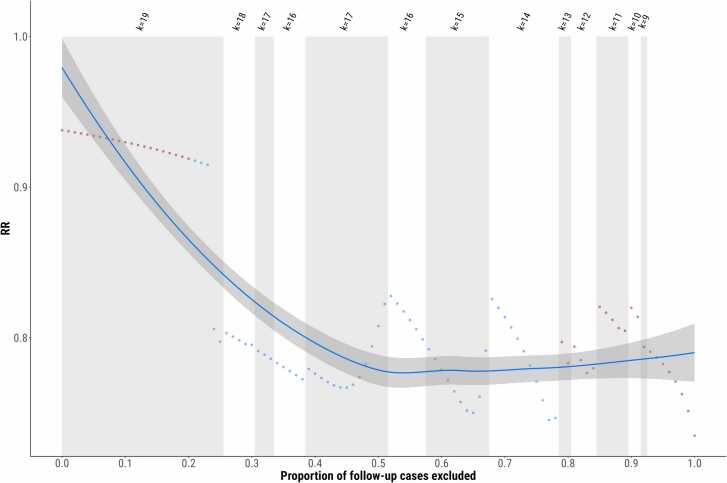
Summary of simulation meta-analytic results. As several included studies reported prevalence at baseline and follow-up, rather than incidence, there were some cases where negative within-group changes occurred (due to individuals dropping out of the study or remitting). We were therefore unable to use conventional meta-analysis. Instead, we simulated the proportion of cases at baseline who did not drop out or remit over the course of follow-up. The resulting number of individuals was subtracted from the cases at follow-up. Those remaining were considered incident cases. The proportion of excluded cases who dropped out or remitted is shown on the x-axis. The corresponding relative risk (RR) from that relevant meta-analysis is shown on the y-axis. RRs below 1 indicate that the intervention group had a lower risk of developing the condition than the control group. As impossible scenarios (where there were more cases than those remaining at follow-up) were excluded from each analysis, the number of studies included in the analysis (k) is shown at the top of the graph. The blue dots represent significant preventive effects, the red dots non-significant effects and the line shows the trend modelled with Loess smoothing and standard errors.

Through our simulations, there was a significant preventive effect in the intervention group compared to control across 58/101 (57 %) of scenarios. k ranged between 9 and 19, RRs ranged between 0.75 and 0.94, lower 95 %CI 0.55 and 0.87, upper 95 %CI 0.93 and 1.15, p-values ranged between 0.007 and 0.246 (Fig. 3; eTable 3).

Individual virtual interventions demonstrated a significant preventive effect in n=18 (18 %) of simulated scenarios (RR=0.43–0.52, lower 95 %CI=0.19–0.29, upper 95 %CI=0.93–0.99, p<0.05). All other intervention types were consistently non-significant (p>0.05; eTable 5).

### 3.2 Heterogeneity, small study effects and risk of bias assessment

The percentage of total variability due to between-study heterogeneity was significant across all scenarios (I^2^=37 %-70 %, p=0.003–0.24). Evidence of small study effects were seen in meta-analyses of prevalence under 83 (91 %) scenarios. k=3 studies were filled in 2 scenarios, k=4 studies were filled in 5 scenarios, k=5 studies were filled in 4 scenarios and k=6 studies were filled in 36 scenarios, k=7 studies were filled in 35 scenarios, k=8 studies were filled in 8 scenarios and k=9 studies in 2 scenarios. All scenarios resulted in non-significant results (p>0.05; eTable 4). Risk of bias assessments showed that only 2 studies (11.1 %) were at low risk of bias, 1 study (5.5 %) had some concerns and the remaining 15 studies (83.3 %) were at high risk of bias (eTable 6–7). High risk of bias was largely due to outcome measurement in studies employing self-report instruments of depression with participants having knowledge of group allocation (13 studies; 86.7 %).

### 3.3 Sensitivity analysis

**Fig. 4:**
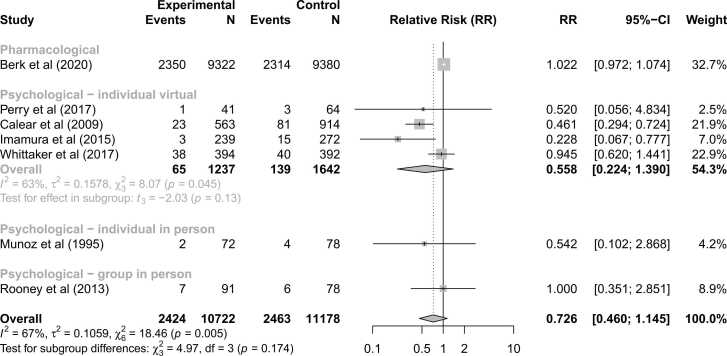
Forest plot for primary sensitivity analysis restricted to studies investigating universal preventive interventions for reducing the incidence of depression.

Overall, when restricting to studies directly reporting incidence, there was no statistically significant effect of universal preventive interventions on reducing the incidence of depression (RR=0.73; 95 %CI: 0.46,1.15; p=0.14; Fig. 4). There were no significant differences in effect sizes between intervention types (Q=4.97, p=0.17) and no intervention type showed a significant change in relative risk compared to the control group (p>0.05; Fig. 4).

There was no significant effect of psychological group, individual, in person or virtual interventions on incidence of depression or prevalence of depression (p>0.05). When analyses were restricted to samples assessed with clinical interviews, there remained no significant effect of interventions on reducing incidence of depression (k=5, RR=0.86–0.99; lower 95 %CI 0.39–0.82, upper 95 %CI 1.07–2.23, p-values ranged between 0.297 and 0.895).

## 4 Discussion

To our knowledge, this is the first study to synthesise the available evidence for the effectiveness of universal preventive interventions for reducing the incidence of psychotic and affective disorders. We found some evidence that universal preventive interventions are effective at reducing the incidence of depression. We did not find strong evidence of a significant preventive effect of universal interventions for reducing the incidence of psychotic or bipolar disorders.

### 4.1 Universal interventions for the prevention of depression

Currently, the available evidence suggests that universal preventive interventions have a significant effect in reducing the incidence of depression. However, this effect was not maintained when restricted to studies that directly measured incidence or those that used more robust diagnostic interviews to measure outcomes. The lack of high quality data resulted in the use of simulation analysis, highlighting the need to improve research quality. 89% of simulated scenarios were associated with significant small study effects, suggesting that these results may be biased towards a preventive effect. This is further emphasised by most studies being considered to be at high risk of bias. Despite the promising nature of these findings, they underline the need for future research into universal prevention, particularly rigorous, high quality, well-powered studies. The majority of the studies included were based on universal psychological and psycho-educational interventions. The only intervention type that had a significant preventive effect were virtual individual psychological interventions but this was only in a small proportion of the simulated scenarios.

These results are similar to previous evidence syntheses, found that online psychological and psychoeducational interventions have a small effect on reducing depressive symptoms in non-depressed and diverse populations, with moderate quality of evidence (Rigabert et al., 2020). An umbrella review (Hoare et al., 2021) found a preventive effect of school-based interventions, which our results did not replicate. However, comparisons between our study and previous evidence syntheses should be made with caution as, unlike in our paper, previous evidence syntheses have assessed the effect of interventions on symptom severity, rather than incidence of depression. Therefore, while this evidence suggests that these interventions may be able to reduce the severity of depressive symptoms in a universal sample, these results are not sufficient to inform their ability to prevent disorder onset.

Regarding other study types, only one study investigated a pharmacological intervention (aspirin) for the prevention of depression (Berk et al., 2020) and did not show a significant preventive effect. Indeed, the challenge for pharmacological interventions is finding a putative molecule with preventive potential (e.g. anti-inflammatory properties of aspirin) without producing adverse effects that outweigh the benefits of the intervention (e.g. an increased risk of haemorrhagic events). The risks associated with adverse events becomes increasingly prominent with universal preventive interventions as the majority of the target population will not develop the outcome (e.g. depression) even in the absence of any intervention. Due to this, any benefit of any future licensed universal preventive interventions, in particular pharmacological interventions, will have to be large to outweigh potential adverse effects.

Even within each intervention type, there can be substantial heterogeneity in the design and implementation of interventions, which can have implications on their effectiveness. More research is needed to identify effective universal preventive interventions for depression and the core features that determine their effectiveness to apply them to other approaches.

### 4.2 Universal interventions for the prevention of bipolar and psychotic disorders

No studies on universal preventive interventions for psychotic and bipolar disorders were identified. This may be due to their relatively low prevalence compared to depression (Perälä et al., 2007) and the long latency period between exposure to predisposing factors and the onset of subsyndromal and/or full-blown symptoms (Hall and Degenhardt, 2008). Investigating universal intervention for these disorders therefore requires large population samples and a long follow-up period, which increases the logistical and financial complexity compared to depression (Szöke et al., 2014).

Due to this, future studies may want to consider investigating universal interventions aiming to reduce the incidence of robust modifiable risk factors or increase the incidence of modifiable protective factors associated with these disorders as proxy measures for reducing their incidence. For example, robust modifiable risk factors for psychosis include the clinical high risk for psychosis state (OR=9.32), cannabis use (OR=3.90) and childhood adversity (OR=2.80) (Arango et al., 2021). For bipolar disorder, there is also a strong association with childhood adversity (OR=2.86) (Arango et al., 2021). In addition to the strength of the association, the prevalence of a risk factor for bipolar disorder or psychosis needs to be considered to estimate the population-level impact of any intervention. This can be demonstrated through the population attributable fraction (PAF): the proportional reduction in population disease that would occur if exposure to a risk factor was eradicated (Dragioti et al., 2022). If childhood adversity and cannabis use were eradicated, the incidence of psychotic disorders would be reduced by 38 % and 10 %, respectively (Dragioti et al., 2022). This is similar to the 10.9–12.3 % prevented if indicated preventive approaches for psychosis were 100% effective (Dragioti et al., 2022, Oliver et al., 2022), emphasising the utility in pursuing complementary approaches. Therefore, effective universal interventions targeting risk factors, like childhood adversity and cannabis use, can be potential proxy targets for reducing the incidence of psychotic and bipolar disorders. This has particularly promise for those factors that are transdiagnostic, like childhood adversity. However, existing evidence for the effectiveness of these interventions has been similarly mixed (Han et al., 2023, Stockings et al., 2016).

The paucity of evidence for effective universal preventive interventions further accentuates the need for complementary approaches. Some authors have argued that universal interventions should be favoured over other forms of prevention (Murray et al., 2021). However, care must be taken to avoid potential misunderstandings. Universal interventions are not intended to replace selective or indicated interventions. Rather, they should be used as complementary measures, particularly where they have been shown to be cost-effective. Given the risk enrichment associated with selective and indicated approaches, there is inherently higher statistical power for randomised controlled trials, increasing the likelihood of demonstrating effective interventions. Selective interventions target individuals with specific exposures to risk factors that increase their vulnerability. Timing of interventions could therefore be important, with interventions potentially being more effective during this vulnerable period. Moreover, in the case of indicated prevention, individuals are help-seeking, presenting with distressing psychopathology (Fusar-Poli et al., 2018). Favouring universal preventive interventions over indicated prevention could be viewed as unethical if it results in reduced care and support for these vulnerable interventions. It is important to recognise that there is currently no concrete evidence of the effectiveness of universal prevention strategies in reducing the incidence of psychotic and affective disorders. It is therefore necessary to continue to use and learn from targeted approaches in order to better identify population-level targets to inform a new generation of universal preventive approaches.

This study has some limitations. Firstly, we found a high risk of bias in the majority of studies. However, this may be explained by the difficulty of blinding psychoeducational and psychological studies. There are creative solutions to this, for example, (Whittaker et al., 2017) investigated a CBT intervention via multimedia messages sent to adolescents in 15 schools in New Zealand. The research team sent identical messages to active and control groups for the first nine days to mitigate against contamination between randomised groups. Secondly, the follow-up period was often relatively short, particularly when considering the peak age of onset of mental disorders (Solmi et al., 2022). Several years of follow-up is costly and logistically difficult, though some studies did accomplish this, with up to 6 years of follow-up (Berk et al., 2020). Third, studies employed a wide range of self-report questionnaires with validated diagnostic cut-offs for depression, with very few studies employing clinical interviews. This limits the validity of depression diagnoses, though we did mitigate against this by performing sensitivity analyses using clinical interview studies only, finding similar results. Fourth, the maximum effect size expected in these studies is small due to the low incidence of these disorders, particularly in the timeframe of follow-up. This creates greater difficulty in demonstrating a clear benefit from interventions (Hoare et al., 2021), requiring larger samples and greater statistical power, so more research is needed.

## 5 Conclusion

In conclusion, we found evidence to support the effectiveness of universal preventive interventions for reducing the incidence of depression. However, while several studies were identified for investigating universal interventions on reducing the incidence of depression, no studies investigating universal interventions on the prevalence or incidence of bipolar disorder or psychosis were identified. Due to the expected small magnitude of effect for universal interventions and small sample size, more high-quality studies with higher statistical power are needed. However, the challenge remains, not only to demonstrate efficacy and safety of universal interventions, but also feasibility of implementation of effective interventions in a real-life context.

## Funding/support

This study is supported by a 10.13039/100004440Wellcome Trust grant (215793/Z/19/Z) to PFP.

## Role of the funder/sponsor

The funder had no role in the design and conduct of the study; collection, management, analysis, and interpretation of the data; preparation, review, or approval of the manuscript; and decision to submit the manuscript for publication.

## Declaration of Competing Interest

GSP has received honoraria from Lundbeck, Menraini and Janssen Cilag outside of the current study. PFP has received research fees from Lundbeck and received honoraria from Lundbeck, Angelini, Menarini and Boehringer Ingelheim outside of the current study.
